# Health-related quality of life in diabetes: The associations of complications with EQ-5D scores

**DOI:** 10.1186/1477-7525-8-18

**Published:** 2010-02-04

**Authors:** Oddvar Solli, Knut Stavem, IS Kristiansen

**Affiliations:** 1 Institute of Health Management and Health Economics, P.O. Box 1089 Blindern, N-0317 Oslo, Norway; 2 Health Services Research Centre, Akershus University Hospital, N-1478 Lørenskog, Norway; 3 Faculty of Medicine, University of Oslo, NO-0316 Oslo, Norway; 4 Institute of Public Health, University of Southern Denmark, DK-5000 Odense, Denmark

## Abstract

**Background:**

The aim of this study was to describe how diabetes complications influence the health-related quality of life of individuals with diabetes using the individual EQ-5D dimensions and the EQ-5D index.

**Methods:**

We mailed a questionnaire to 1,000 individuals with diabetes type 1 and 2 in Norway. The questionnaire had questions about socio-demographic characteristics, use of health care, diabetes complications and finally the EQ-5D descriptive system. Logistic regressions were used to explore determinants of responses in the EQ-5D dimensions, and robust linear regression was used to explore determinants of the EQ-5D index.

**Results:**

In multivariate analyses the strongest determinants of reduced MOBILITY were neuropathy and ischemic heart disease. In the ANXIETY/DEPRESSION dimension of the EQ-5D, "fear of hypoglycaemia" was a strong determinant. For those without complications, the EQ-5D index was 0.90 (type 1 diabetes) and 0.85 (type 2 diabetes). For those with complications, the EQ-5D index was 0.68 (type 1 diabetes) and 0.73 (type 2 diabetes). In the linear regression the factors with the greatest negative impact on the EQ-5D index were ischemic heart disease (type 1 diabetes), stroke (both diabetes types), neuropathy (both diabetes types), and fear of hypoglycaemia (type 2 diabetes).

**Conclusions:**

The EQ-5D dimensions and the EQ-5D seem capable of capturing the consequences of diabetes-related complications, and such complications may have substantial impact on several dimensions of health-related quality of life (HRQoL). The strongest determinants of reduced HRQoL in people with diabetes were ischemic heart disease, stroke and neuropathy.

## Background

Diabetes is a chronic disease with serious short-term and long-term consequences for the afflicted. The total number of individuals with diabetes worldwide is projected to rise from about 170 million in 2000 to about 370 million in 2030 [1]. In the long term, diabetes causes microvascular complications (e.g. retinopathy and neuropathy) and macrovascular complications (e.g. myocardial infarction, angina pectoris and stroke). In addition to diabetes-related complications, episodes of hypoglycaemia, fear of hypoglycaemia, change in life style and fear of long term consequences may lead to reduced health-related quality of life (HRQoL). In fact, individuals with diabetes have reduced HRQoL compared with those without diabetes in the same age group [2,3], and their HRQoL decreases with disease progression and complications [4,5].

There are three main approaches to describe and measure HRQoL: Disease-specific instruments, generic instruments and utility instruments. Numerous disease-specific HRQoL measures exist for diabetes, and these score HRQoL on ordinal scales [6-8]. Generic instruments such as the Short Form 36 (SF-36) are also used [9]. In multi-attribute utility instruments (MAU), such as the EQ-5D [10], 15D [11], Health Utility index (HUI) [12,13] and SF-6D [14], respondents indicate levels of health problems on a number of dimensions of health. These values are translated into a zero-one scale where zero denotes death and one perfect health. Some utility instruments allow for negative values, meaning that some health states are considered worse than death. Preference-based methods such as the time trade-off method (TTO) [15], standard gamble (SG) or the visual analogue scale (VAS) may be used to develop translation algorithms. When the HRQoL weight is multiplied with duration (years, months, duration of effect, expected remaining life years) the product is denoted QALY (quality-adjusted life years) [16]. QALYs can be calculated for different patient groups to compare for example effectiveness of treatment, enabling health improvements and life extensions to be captured in one single variable.

EQ-5D [10] is a MAU instrument with five dimensions (MOBILITY, SELF-CARE, USUAL ACTIVITIES, PAIN/DISCOMFORT and ANXIETY/DEPRESSION) and three levels on each dimension, and has previously been used in populations with diabetes [17]. EQ-5D has been used extensively in economic evaluation, and is recommended for use in cost-effectiveness analyses by institutions such as the National Institute for Clinical Excellence (NICE) in the UK and the Health Care Insurance Board in the Netherlands. Therefore, researchers working with economic evaluation, government agencies and the pharmaceutical industry need easy access to utility data for different types of patients.

Against this background the aim of this study was three-fold:

• To use the five individual EQ-5D dimensions to describe some aspects of HRQoL in a group of people with diabetes.

• To investigate the impact of self-reported diabetes-related complications on the EQ-5D dimension scores.

• To investigate determinants of EQ-5D index in order to offer researchers utility data for individuals with diabetes.

## Methods

The data in this study stem from a Norwegian survey of people with diabetes in 2006. A questionnaire was developed and piloted among health care professionals, including physicians with diabetes expertise and the county leaders of the Norwegian Diabetes Association (NDA). The latter group served as representatives of the target group. The study was approved by the Regional Ethics Committee and the Norwegian Data Inspectorate.

The seven-page questionnaire captured background variables such as age, gender, location, income in Norwegian Kroner (NOK), smoking habits, height, weight, as well as diabetes-specific variables such as diabetes-related health complications and use of health services. Finally, respondents were presented with eight diabetes-specific HRQoL questions and an approved Norwegian translation of the EQ-5D descriptive system. EQ-5D responses were translated into EQ-5D index utilities using the UK TTO tariff [18].

The questionnaire was mailed to a sample of members of the Norwegian Diabetes Association. A large proportion of the individuals with type 1 diabetes in Norway are members of the NDA, while only a minority of those with type 2 diabetes are members. After excluding individuals under the age of 18 years and those without diabetes, such as health care workers and others with an interest in diabetes, the NDA drew a random sample of 1,000 members. Non-respondents were followed up twice. The last follow up was accompanied by a letter from the NDA explaining the importance of insight in diabetes and encouraging response.

### Data analyses

For descriptive statistics, we used means, proportions and standard deviations. Determinants of EQ-5D dimension values were analysed by logistic regression. For all 5 dimensions level 2 and 3 on the EQ-5D dimensions were merged and thus dichotomized to "no problem" or "some or extreme problem". We performed separate regressions for type 1 and type 2 diabetes.

The EQ-5D index was analysed with a linear OLS regression model. The Breusch-Pagan test and plotting residuals versus fitted values showed that heteroscedasticity was present both for type 1 and type 2 diabetes. Therefore, we applied White's robust variance estimators.

The data were complete except for the covariates "Fear of hypoglycaemia" (13% missing), "Limitations at work" (23% missing) and "Limitations socially" (10% missing). Missing values were therefore imputed with regressions based on 15 independent variables (sex, age, weight, height and 11 diabetes-related complications). We used the impute function in STATA, which runs regressions by simple best-subset linear regression, looking at the pattern of missing values in the predictors.

We tested the covariates age and body mass index first as dummy variables divided in quartiles and second as continuous variables.

We chose covariates for the models based on input from health care professionals and representatives from academia. In the binary regressions the selected variables are considered plausible to be linked with the dimension analysed. In addition to "Sex" and "age", all direct medical complications were included in all dimensions except "Proteinuria". We believe this covariate is likely only to remind the individuals of lurking complications and should thus only impact the ANXIETY/DEPRESSION dimension. The variable "Impaired vision" is in our view not likely to directly cause pain or discomfort and is not included in regression of the PAIN/DISCOMFORT dimension. Emotional impact of impaired vision should be captured in the ANXIETY/DEPRESSION dimension.

In both the logistic binary and the linear regressions full sets of the selected covariates were kept throughout the analysis in order to provide variables with both significant and non-significant impact on the covariates. For the linear regression this would provide a full set of results which may be used by other analysts in decision analytic modelling.

All analyses were performed in STATA/SE 10.0 (Stata Corp, College Station, TX, USA).

## Results

### Sample characteristics

Of the total 1,000 eligible individuals with diabetes, 17 were excluded because they had died (n = 4) or had unknown address (n = 13). Two persons declined to participate. In total 598 of those eligible returned the questionnaire, of which 521 were complete and could be used in further analysis (response rate 53%). Among non-respondents, 51% were female compared with 47% among respondents.

Among the 521 respondents, 165 reported having type 1 diabetes (53% female), and 356 type 2 diabetes (44% female) (Table 1). Further descriptive statistics about demographics, risk, factors for complications, medication and complications are shown in Table 1.

**Table 1:** Characteristics of the respondents according to diabetes type, number (%), unless otherwise specified

	Type 1	Type 2
*n*	*165*	*356*
***Demographics***		
Sex, female	87 (53)	157 (44)
Age, mean (SD)	47.0 (14.9)	64.0 (11.7)
Annual family income (1000 NOK), mean (SD)	666 (908)	713 (3051)
		
***Complication risk factors***		
Diabetes duration (years), mean (SD)	22.1 (14.2)	10.0 (8.1)
Current smoking	47 (29)	62 (18)
Daily smoker	22 (14)	42 (12)
Occasional smoker	25 (15)	20 (6)
Previous smokers	86 (55)	200 (61)
Body mass index, kg/m^2 ^, mean (SD)	25.8 (4.8)	28.9 (5.1)
		
***Medication***		
Number of oral antidiabetic agents		
0	159 (96)	103 (29)
1	4 (2)	149 (42)
2	2 (1)	87 (24)
3	---	16 (5)
4	---	1 (0.3)
Insulin		
Short-acting insulin	152 (92)	68 (19)
Long-acting insulin	103 (62)	98 (28)
Insulin glargine (Lantus) or insulin detemir (Levemir)	51 (31)	11 (3)
Antihypertensives	52 (33)	217 (63)
Cholesterol lowering drug	45 (28)	205 (59)
		
***Self-reported complications***		
Impaired vision	31 (19)	51 (14)
Myocardial infarction	4 (2)	38 (11)
Angina	10 (6)	27 (8)
Reduced kidney function (Proteinuria)	15 (9)	24 (7)
Kidney transplant	1 (1)	2 (1)
Foot ulcer	6 (4)	13 (4)
Amputation	2 (1)	1 (0.3)
Stroke	5 (3)	19 (5)
Neuropathy	12 (7)	17 (5)
Other	37 (22)	53 (15)
None	79 (47)	161 (45)

### Health-related quality of life

In total 10% of those with type 1 diabetes had problems with MOBILITY as judged from the EQ-5D, 3% with SELF-CARE, 19% with USUAL ACTIVITIES, 34% with PAIN/DISCOMFORT and 35% with ANXIETY/DEPRESSION (Table 2). For Type 2 diabetes the numbers were 26%, 6%, 25%, 45% and 33%, respectively. The mean EQ-5D index score was 0.83 (SD 0.24) in type 1 diabetes and 0.81 (SD 0.22) in type 2 (*p *= 0.32). The proportion of type 2 diabetes patients with fear of hypoglycaemia was 50% among those on insulin and 26% among the others.

**Table 2:** Distribution of levels of perceived problem in each of the dimensions of the EQ-5D descriptive system, according to diabetes type

	Type 1 (n = 165)			Type 2 (n = 356)		
	
	Level of perceived problem, %					
**Dimension**	1*	2*	3*	1*	2*	3*
Mobility	90	10	0	74	26	0
Self-care	97	3	0	94	6	0
Usual activities	81	18	1	74	24	1
Pain/discomfort	65	29	5	56	41	4
Anxiety/depression	65	32	3	67	30	3

* Level 1 implies no problem, 2 moderate problem, 3 severe problem

For individuals without any reported complications, the mean EQ-5D index scores were 0.90 for those with type 1 diabetes and 0.85 for those with type 2 (Table 3). The presence of one complication decreased values to 0.76 and 0.80, respectively. With two or more diabetes-related complications the values were 0.55 and 0.64, respectively.

**Table 3:** Mean EQ-5D index utility values with and without diabetes-related complications

	Type 1 diabetes			Type 2 diabetes		
	
Number of complications	EQ-5D index	95% CI	n	EQ-5D index	95% CI	n
0	0.90	0.88 - 0.93	111	0.85	0.82 - 0.87	241
1	0.76	0.66 - 0.86	35	0.80	0.75 - 0.85	68
≥ 2	0.55	0.37 - 0.73	19	0.64	0.56 - 0.71	47
Any complication	0.68	0.59 - 0.77	54	0.73	0.69 - 0.78	115
All patients	0.83	0.79 - 0.87	165	0.81	0.79 - 0.83	356

### Regression analyses

In the binary logistic regressions of type 1 diabetes on EQ-5D dimension responses (Table 4), ischemic heart disease, foot ulcer, neuropathy, body mass index and receiving help from others were statistically significant determinants for reporting problems in the MOBILITY dimension. None of the covariates had impact on the SELF-CARE dimension. Disability pension and limitations at work had an impact on the USUAL ACTIVITIES dimension. Age, ischemic heart disease and neuropathy had an impact on the PAIN/DISCOMFORT dimension, and age, impaired vision, ischemic heart disease, neuropathy and fear of hypoglycaemia had an impact on the ANXIETY/DEPRESSION dimension.

**Table 4:** Binary multivariate logistic regression of responses to the EQ-5D items in type 1 diabetics, odds ratios (95% CI)

	EQ-5D dimensions				
	
	Mobility	Self-care	Usual activities	Pain/discomfort	Anxiety/depression
Sex (male = 0, female = 1)	0.63 (0.14 - 2.74)	0.25 (0.01 - 5.15)	0.67 (0.22 - 2.03)	0.45 (0.20 - 1.03)	1.12 (0.50 - 2.51)
Age (in 10 years)	1.33 (0.78 - 2.25)	1.37 (0.55 - 3.43)	0.93 (0.61 - 1.40)	1.36 (1.04 - 1.77)*	0.72 (0.55 - 0.94)*
Impaired vision (no = 0, yes = 1)	3.00 (0.53 - 16.85)	12.11 (0.49 - 297.88)	0.28 (0.07 - 1.15)	-----	4.60 (1.57 - 13.46)**
Ischemic heart disease (no = 0, yes = 1)	11.72 (2.02 - 68.09)**	1.24 (0.05 - 31.42)	4.15 (0.73 - 23.64)	5.84 (1.29 - 26.40)*	6.82 (1.34 - 34.75)*
Proteinuria (no = 0, yes = 1)	-----	-----	-----	-----	0.47 (0.09 - 2.47)
Foot Ulcer (no = 0, yes = 1)	13.33 (1.33 - 133.29)*	6.20 (0.17 - 221.73)	10.04 (0.80 - 126.22)	3.24 (0.47 - 22.43)	1.06 (0.16 - 6.96)
Stroke (no = 0, yes = 1)	0.47 (0.02 - 8.99)	17.37 (0.49 - 610.92)	1.24 (0.09 - 16.83)	10.66 (0.75 - 152.16)	1.14 (0.13 - 10.21)
Neuropathy (no = 0, yes = 1)	7.17 (1.22 - 42.03)*	5.86 (0.41 - 83.43)	6.96 (1.45 - 33.44)	27.13 (3.13 - 235.07)**	4.61 (1.05 - 20.21)*
Body mass index (kg/m^2 ^)	1.15 (1.02 - 1.30)*	-----	-----	-----	-----
Disability pension (no = 0, yes = 1)	-----	-----	4.64 (1.33 - 16.18)*	-----	-----
Number of hospital admissions during previous 6 months	-----	-----	-----	-----	1.22 (0.58 - 2.53)
Receives help from others (no = 0, yes = 1)	10.04 (2.03 - 49.69)**	10.28 (0.61 - 173.34)	1.90 (0.50 - 7.22)	-----	-----
Hypoglycaemia index#	-----	-----	-----	1.59 (0.87 - 2.89)	1.29 (0.71 - 2.33)
Fear of hypoglycaemia## (small = 0, large = 1)	-----	-----	-----	-----	3.98 (1.78 - 8.93)**
Limitations at work## (small = 0, large = 1)	-----	-----	13.20 (3.38 - 51.53)***	-----	-----
Limitations socially## (small = 0, large = 1)	-----	-----	1.87 (0.65 - 5.37)	-----	-----
Log likelihood	-28.08	-9.96	-51.46	-85.06	-85.30

* p < 0.05, **p < 0.01, ***p < 0.001

Cells with dotted line indicate that the variable was not included in the model.

# Self reported episodes of hypoglycaemia, with 4 levels of severity (level 1 = hypoglycaemia cured with the intake of for example fluids containing sugar, no help from other required, level 2 = hypoglycaemia cured with the intake of for example fluids containing sugar, help from others required, level 3 = hypoglycaemia with help from doctor required (no hospital admission), level 4 = hypoglycaemia resulting in hospital admission), then added with severity weights (level 1 × 1, level 2 × 2, level 3 × 3, level 4 × 4) and finally divided in 3 groups 0, 1-11 and 12 to max

## Self reported on a scale from 1 to 5 (1 = not at all, 5 = very much), recoded to 2 levels (> and < than 2.5 due to imputed values having values with decimals)

For type 2 diabetes (Table 5), age, impaired vision, stroke, neuropathy, body mass index and receiving help from others were statistically significant determinants of MOBILITY. Receiving help from others for SELF-CARE, sex, stroke, disability pension, receiving help from others and limitations at work were associated with USUAL ACTIVITIES. Ischemic heart disease, neuropathy and hypoglycaemia had an impact on PAIN/DISCOMFORT. Age, foot ulcers, number of hospital admissions during the previous 6 months and fear of hypoglycaemia were associated with ANXIETY/DEPRESSION scores.

**Table 5:** Binary multivariate logistic regression of responses to the EQ-5D items in type 2 diabetics, odds ratios (95% CI)

	EQ-5D dimensions				
	
	Mobility	Self-care	Usual activities	Pain/discomfort	Anxiety/depression
Sex (male = 0, female = 1)	0.68 (0.38 - 1.21)	0.59 (0.23 - 1.54)	0.47 (0.25 - 0.88)*	0.82 (0.53 - 1.27)	0.91 (0.54 - 1.52)
Age (in 10 years)	1.36 (1.03 - 1.80)*	0.83 (0.55 - 1.25)	1.34 (1.00 - 1.80)	1.03 (0.83 - 1.24)	0.78 (0.62 - 0.99)*
Impaired vision (normal = 0, reduced = 1)	2.96 (1.44 - 6.10)**	2.29 (0.77 - 6.75)	0.89 (0.39 - 2.04)	-----	1.46 (0.71 - 3.01)
Ischemic heart disease (no = 0, yes = 1)	1.97 (0.91 - 4.25)	1.77 (0.54 - 5.86)	1.14 (0.48 - 2.71)	2.51 (1.27 - 4.97)**	1.15 (0.53 - 2.50)
Proteinuria (no = 0, yes = 1)	-----	-----	-----	-----	0.42 (0.14 - 1.29)
Foot Ulcer (no = 0, yes = 1)	0.32 (0.07 - 1.39)	0.73 (0.11 - 4.67)	2.11 (0.48 - 9.39)	2.18 (0.54 - 8.79)	7.00 (1.53 - 31.97)*
Stroke (no = 0, yes = 1)	3.50 (1.13 - 10.82)*	1.45 (0.23 - 9.13)	4.48 (1.38 - 14.59)*	1.99 (0.72 - 5.54)	2.14 (0.69 - 6.62)
Neuropathy (no = 0, yes = 1)	12.07 (3.30 - 44.12)***	2.74 (0.57 - 13.25)	3.08 (0.84 - 11.26)	Predicts perfectly#	1.29 (0.40 - 4.16)
Body mass index (kg/m^2 ^)	1.12 (1.05 - 1.19)***	-----	-----	-----	-----
Disability pension (no = 0, yes = 1)	-----	-----	2.38 (1.20 - 4.69)*		-----
Number of hospital admissions during previous 6 months	-----	-----	-----	-----	1.87 (1.14 - 3.07)*
Receives help from others (no = 0, yes = 1)	5.85 (3.00 - 11.38)***	6.95 (2.58 - 18.73)***	4.67 (2.21 - 9.87)***	-----	-----
Hypoglycaemia index##	-----	-----	-----	1.68 (1.13 - 2.49)*	1.08 (0.70 - 1.68)
Fear of hypoglycaemia### (small = 0, large = 1)	-----	-----	-----	-----	5.76 (3.36 - 9.87)***
Limitations at work### (small = 0, large = 1)	-----	----	6.95 (3.56 -13.56)***	-----	-----
Limitations socially### (small = 0, large = 1)	-----	----	1.33 (0.67 - 2.62)	-----	-----
Log likelihood	-156.08	-66.13	-136.85	-232.10	-187.32

* p < 0.05, **p < 0.01, ***p < 0.001

# All patients reporting neuropathy also reports having problems in the PAIN/DISCOMFORT dimension of the EQ-5D.

Cells with dotted line indicate that the variable was not included in the model.

## Self reported episodes of hypoglycaemia, with 4 levels of severity (level 1 = hypoglycaemia cured with the intake of for example fluids containing sugar, no help from other required, level 2 = hypoglycaemia cured with the intake of for example fluids containing sugar, help from others required, level 3 = hypoglycaemia with help from doctor required (no hospital admission), level 4 = hypoglycaemia resulting in hospital admission), then added with severity weights (level 1 × 1, level 2 × 2, level 3 × 3, level 4 × 4) and finally divided in 3 groups 0, 1-11 and 12 to max

### Self reported on a scale from 1 to 5 (1 = not at all, 5 = very much), recoded to 2 levels (> and < than 2.5 due to imputed values having values with decimals)

In the linear regression of the EQ-5D index for type 1 diabetes, presence of ischemic heart disease had a negative impact (-0.181), along with stroke (-0.291), neuropathy (-0.358), receiving disability pension (-0.111) and social limitations (-0.107) (Table 6). For type 2 diabetes the following conditions had a negative impact (Table 6): stroke (-0.135), neuropathy (-0.187), disability pension (-0.100), receiving help from others (-0.123), fear of hypoglycaemia (-0.078) and limitations at work (-0.087). For both diabetes types we tested for interactions, but found none. We found no effect of age or body mass index in the linear regressions whether age and BMI were entered as one continuous variable or as dummy variables.

**Table 6:** Linear multivariate regression of EQ-5D index, according to diabetes type

	Type 1		Type 2	
N	165		356	
	Coefficient (95% CI)	P > |t|	Coefficient (95% CI)	P > |t|
Constant	1.092 (0.921 to 1.263)	<0.001	0.990 (0.787 to 1.193)	<0.001
Sex (male = 0, female = 1)	0.041 (-0.023 to 0.105)	0.210	0.024 (-0.016 to 0.064)	0.240
Age (in 10 years)	-0.003 (-0.022 to 0.016)	0.749	0.0004 (-0.017 to 0.017)	0.967
Impaired vision (no = 0, yes = 1)	-0.063 (-0.169 to 0.044)	0.245	-0.012 (-0.074 to 0.051)	0.711
Ischemic heart disease (no = 0, yes = 1)	-0.181 (-0.331 to -0.031)	0.019	-0.037 (-0.103 to 0.030)	0.276
Proteinuria (no = 0, yes = 1)	0.089 (-0.036 to 0.215)	0.161	0.043 (-0.019 to 0.106)	0.174
Foot Ulcer (no = 0, yes = 1)	-0.083 (-0.271 to 0.105)	0.383	-0.016 (-0.134 to 0.101)	0.783
Stroke (no = 0, yes = 1)	-0.291 (-0.475 to -0.108)	0.002	-0.135 (-0.247 to -0.023)	0.018
Neuropathy (no = 0, yes = 1)	-0.358 (-0.535 to -0.180)	<0.001	-0.187 (-0.316 to -0.057)	0.005
Body mass index (kg/m^2 ^)	-0.004 (-0.008 to 0.001)	0.123	-0.002 (-0.007 to 0.002)	0.307
Disability pension (no = 0, yes = 1)	-0.111 (-0.191 to -0.030)	0.008	-0.100 (-0.153 to -0.046)	<0.001
Number of hospital admissions during previous 6 months	0.003 (-0.042 to 0.049)	0.880	-0.028 (-0.076 to 0.020)	0.255
Receives help from others (no = 0, yes = 1)	-0.090 (-0.217 to 0.037)	0.166	-0.123 (-0.185 to -0.060)	<0.001
Hypoglycaemia index#	-0.023 (-0.071 to 0.025)	0.337	-0.004 (-0.039 to 0.032)	0.839
Fear of hypoglycaemia## (small = 0, large = 1)	-0.021 (-0.073 to 0.031)	0.432	-0.078 (-0.129 to -0.028)	0.003
Limitations at work## (small = 0, large = 1)	-0.023 (-0.089 to 0.043)	0.494	-0.087 (-0.148 to -0.025)	0.006
Limitations socially## (small = 0, large = 1)	-0.107 (-0.188 to -0.026)	0.010	-0.002 (-0.049 to 0.046)	0.944

# Self reported episodes of hypoglycaemia, with 4 levels of severity (level 1 = hypoglycaemia cured with the intake of for example fluids containing sugar, no help from other required, level 2 = hypoglycaemia cured with the intake of for example fluids containing sugar, help from others required, level 3 = hypoglycaemia with help from doctor required (no hospital admission), level 4 = hypoglycaemia resulting in hospital admission), then added with severity weights (level 1 × 1, level 2 × 2, level 3 × 3, level 4 × 4) and finally divided in 3 groups 0, 1-11 and 12 to max ## Self reported on a scale from 1 to 5 (1 = not at all, 5 = very much), recoded to 2 levels (> and < than 2.5 due to imputed values having values with decimals)

## Discussion

In this study, individuals with diabetes-related complications had reduced HRQoL, though the impact on HRQoL was somewhat different for type 1 and type 2 diabetes. Stroke and neuropathy had a negative impact on overall HRQoL in both types of diabetes, while ischemic heart disease and social limitations had an impact on those with type 1 diabetes, and fear of hypoglycaemia and limitations at work had an impact on those with type 2 diabetics. Individuals with type 1 diabetes reported more problems than those with type 2 in the PAIN/DISCOMFORT and ANXIETY/DEPRESSION dimensions, while in the MOBILITY, SELF-CARE and USUAL ACTIVITIES dimensions it was opposite. In spite of the limited descriptive system of the EQ-5D, the instrument still captures the impact of several diabetes complications both with respect to each of the dimensions and the EQ-5D index, and therefore individual EQ-5D dimensions seem well suited to capture most diabetes-related complications.

In a 2009 review of quality of life measurement in adults with diabetes [19] the authors claim that the EQ-5D measures quality of health and not quality of life and that the EQ-5D lacks responsiveness for use in diabetes. The authors state that while the EQ-5D may capture differences due to diabetes related complications it will not necessarily be able to capture differences across treatment regimens. This is because the extent to which a given treatment is considered flexible or convenient will not affect quality of health but may affect aspects of quality of life, such as social or working life. The authors suggest using diabetes-specific instruments or a different generic instrument more sensitive to differences between treatments. Our results show that while both the individual dimensions of the EQ-5D and the EQ-5D index are able to capture typical diabetes-related complications, the subgroups without complications reported surprisingly high EQ-5D index values. This may indicate that the EQ-5D instrument was not able to capture important non-health aspects of quality of life, as claimed in the review [19]. Because the EQ-5D instrument is not diabetes specific, lowered scores may reflect the impact of unrelated comorbidity. A condition specific instrument such as the ADDQoL may differentiate better between diabetes related complications and unrelated comorbidity [19].

In the present study, the finding that individuals with type 1 diabetes reported better HRQoL than those with type 2 can be explained by the younger age of the former group. The opposite was observed in subgroups with complications, and it seems as if diabetic complications had more impact on HRQoL in type 1 diabetes than type 2. A possible explanation is that complications are likely to have a greater impact on the health of people with type 1 diabetes precisely because they are younger, i.e. have less comorbidity and have not adjusted to the idea of accepting lesser health. The differences could also be explained by the fact that this younger subgroup has responsibilities such as work and family as well as relationship issues that are not found in the older subgroup with type 2 diabetes.

In the UKPDS 37 study [20] individuals with type 2 diabetes and no complications had a mean EQ-5D index value of 0.83, compared with 0.85 in our study. In type 2 diabetes with complications, our observed EQ-5D index value (0.73) was equal to that of the UKPDS 37 study. Taking into account that patient characteristics were similar in the UKPDS and our study, UK diabetes studies may be transferable to the Norwegian setting. In the UKPDS 37 study the EQ-5D detected significant differences between people with and without macrovascular complications, but not microvascular complications or using different treatment regimens. In our study the microvascular complication neuropathy had impact on the individual EQ-5D dimensions and on the EQ-5D index.

In another UK study [21] of individuals with type 2 diabetes, the change in utility associated with fear of hypoglycaemia was relatively small compared with the disutility for serious diabetic complications such as neuropathy. Similarly, in our study fear of hypoglycaemia caused a reduction in utility of 0.021 (type 1 diabetes) and 0.078 (type 2), while the disutility of neuropathy was larger with 0.358 (type 1 diabetes) and 0.187 (type 2 diabetes). We have no clear explanation why our results indicate a lower impact on HRQoL of fear of hypoglycaemia in individuals with type 1 diabetes than those with type 2 diabetes. Fear of hypoglycaemia may not affect HRQoL particularly (e.g. has little impact on pain or mobility) but it can affect aspects of more general quality of life (e.g. independence, spontaneity, ability to work, enjoyment of leisure activities).

In a US review [22] of body weight and HRQoL in type 2 diabetes, the authors found decreasing HRQoL with increasing body weight in all included studies. When adjusting for other explanatory variables, we observed no significant impact of BMI on HRQoL.

A subgroup of individuals with unspecified type diabetes (n = 117) in a Swedish general population EQ-5D study [23], also using the UK tariff, reported a higher frequency of problems in all dimensions of the EQ-5D, than in both diabetes categories in our study. Further, the respondents in the study reported a lower mean EQ-5D index (0.74) than we observed in both type 1 and type 2 diabetes.

Some limitations of the present study should be noted. The respondents in the survey may not be representative of the population with diabetes. In particular, bias may arise because sicker and older persons with type 2 diabetes did not respond to the survey. A large proportion of individuals with type 1 diabetes in Norway (about 20,000) are members of the NDA while only a smaller proportion of the type 2 (about 100,000) are members of this organization. Clearly, our study does not capture HRQoL in undiagnosed diabetes patients. In line with other patient surveys, we had 47% non-response. We have no information on non-respondents except for sex (based on non-respondents first names), and here there was little difference between responders and non-responders.

It is important to be aware that because the EQ-5D instrument is no diabetes specific it may reflect problems related to other conditions. Our study was performed at one point in time, and fluctuations are likely to occur if HRQoL was measured at multiple points in time. The observed associations are not necessarily causal. Further they are limited by the lack of serial observations. Furthermore, the limited sample size, especially for type 1 diabetes may limit the power for some of the comparisons of presence or absence of complications.

Note that despite the index score being a function of the score in the dimensions a significant impact on linear regression of the index does not necessarily imply a significant impact on one or more of the dimensions. This is the case for the covariate "stroke" which is significant in both types of diabetes in the linear regression by not significant in any of the dimensions in the type 1 diabetes group.

Lacking a Norwegian EQ-5D tariff we used the UK tariff, based on TTO [18]. This tariff is probably the most commonly used EQ-5D tariff globally, and quite similar to the Danish one [24]. Also, one small Norwegian study indicates that UK and Norwegian values are quite similar [25].

## Conclusions

In this sample of people with diabetes, the individual EQ-5D dimensions were able to capture diabetes-related complications. The results show that such complications may have an impact on many dimensions of health-related quality of life, and the impact may be substantial. The strongest determinants of reduced HRQoL, as assessed with the EQ-5D index, were ischemic heart disease, stroke and neuropathy. The complexity of the disease means that several dimensions need to be considered when priorities are set for diabetes interventions.

## Competing interests

The authors declare that they have no competing interests.

## Authors' contributions

OS developed the study design, collected data, performed the analyses and drafted the manuscript. KS and ISK provided inputs on design and revised the manuscript during the writing. All authors read and approved the final manuscript.
